# Predicting the Mortality Benefit of CT Screening for Second Lung Cancer in a High-Risk Population

**DOI:** 10.1371/journal.pone.0165471

**Published:** 2016-11-02

**Authors:** C. Matthew Kinsey, Katharine L. Hamlington, Jacqueline O’Toole, Renee Stapleton, Jason H. T. Bates

**Affiliations:** 1 Pulmonary and Critical Care Division, University of Vermont College of Medicine, Burlington, Vermont, United States of America; 2 Department of Medicine, University of Vermont College of Medicine, Burlington, Vermont, United States of America; Peking University People's Hospital, CHINA

## Abstract

Patients who survive an index lung cancer (ILC) after surgical resection continue to be at significant risk for a metachronous lung cancer (MLC). Indeed, this risk is much higher than the risk of developing an ILC in heavy smokers. There is currently little evidence upon which to base guidelines for screening at-risk patients for MLC, and the risk-reward tradeoffs for screening this patient population are unknown. The goal of this investigation was to estimate the maximum mortality benefit of CT screening for MLC. We developed a computational model to estimate the maximum rates of CT detection of MLC and surgical resection to be expected in a given population as a function of time after resection of an ILC. Applying the model to a hypothetical high-risk population suggests that screening for MLC within 5 years after resection of an ILC may identify only a very small number of treatable cancers. The risk of death from a potentially resectable MLC increases dramatically past this point, however, suggesting that screening after 5 years is imperative. The model also predicts a substantial detection gap for MLC that demonstrates the benefit to be gained as more sensitive screening methods are developed.

## Introduction

Lung cancer is the leading cause of cancer-related death in the United States [1] and has a treatment success that is dramatically affected by stage at detection [2]. Unfortunately, patients who survive their initial (index) cancer (ILC) continue to be at significant risk for a second primary (metachronous) lung cancer (MLC). MLC has traditionally been defined as a new cancer with a different histology from the initial cancer, or if of the same histology, occurring in a different lobe or lung following an at least two years cancer-free interval and without intervening carcinoma in lymph nodes or extrapulmonary metastases at the time of diagnosis. While the risk of a recurrent cancer diminishes over time the risk of MLC remains stable.[3] Indeed, the risk of developing MLC in patients who have undergone potentially curative surgery for an ILC is higher than the risk of ILC in heavy smokers [3,4].

Several prior studies have reported good outcomes for treatment of MLC [5,6]. Based on the mortality benefit associated with annual CT screening of current or former smokers at risk for an ILC [7,8], the wisdom of annual CT screening for MLC in lung cancer survivors might seem obvious. However, data documenting a benefit are limited [9,10], and there are potentially greater risks with frequent screening. Approximately 50% of CT scans performed after treatment of an ILC result in an abnormality requiring investigation as a possible cancer as determined by the treating clinician, a rate twice as high as for smokers without a history of lung cancer[11]. Thus, performing CT scans to detect MLC carries a substantial risk of procedural morbidity and mortality that must be weighed against an unknown benefit.

Accordingly, the goal of our study was to calculate the maximum reductions in mortality that are achievable by CT screening for MLC as a best-case scenario, without the competing risk of recurrent lung cancer. We made these calculations using a novel computational model that allows us to estimate the maximum possible rates of MLC detection and cure to be expected in a given population as a function of time after resection of an ILC.

## Materials and Methods

We predicted the natural history of MLC by simulating the stochastic appearance of an ILC and a subsequent MLC in a population of individuals drawn randomly from specified distributions of age and smoking history (Table 1). The efficacy of CT screening for MLC was then determined in a Monte-Carlo simulation based on the probability of detection of each tumor according to its size.

**Table 1:** Characteristics of the Simulated Populations of At-risk Individuals.

Parameter	Value
Smoking intensity, *n*_0_^a^	20 (5) cigarettes/day
Smoking start age, *y*_*start*_^a^	16 (2) years
Smoking quit age, *y*_*quit*_^a^	80 (5) years
Tumor doubling time, *T*_*d*_^a^	190 (45) days
Minimum age at first screening, *y*_*1*_	55 years
Maximum age at first screening, *y*_*2*_	75 years

^a^ mean (SD).

### Model of Lung Cancer Growth

We assume that a cancerous lung tumor begins with the appearance of a single malignant cell that then proliferates exponentially with a doubling time of *T*_*d*_ days. If a 1 ml tumor contains 10^9^ malignant cells [12], a single cancerous cell has a volume of 10^−9^ ml. Thus, a tumor that started *m* years ago has a volume, *V*_*tumor*_, of

$$V_{tumor}\left(m\right)=10^{-9}\text{exp}\left(\frac{365m\text{ln}\left(2\right)}{T_{d}}\right).$$ (1)

The probability, *P*_1_, that a randomly selected individual in the population has just developed a single cancerous cell in their lungs is a monotonically increasing function of both their age in years (*y*) and their smoking exposure, which are the strongest risk factors for lung cancer.[13] We initially derived an expression for *P*_1_ based on the empirical equation developed by Lubin et al. [14] for the odds ratio, *O*_*R*_, of dying from lung cancer when smoking begins at a specified age and continues at a fixed intensity until the time of death:

$$O_{R}=1+\beta d\text{exp}\left(\varphi_{1}\text{ln}\left(n\right)+\varphi_{2}{\text{ln}}^{2}\left(n\right)\right),$$ (2)

where *n* is the smoking intensity in cigarettes per day, *d* is pack-years (product of the number of 20-cigarette packs smoked per day and the number of years of smoking), and *β*, *φ*_1_, and *φ*_2_ are scaling parameters. To account for the decreasing risk of death from lung cancer associated with smoking cessation eq 2 was modified. The effective smoking intensity decreases hyperbolically with age after the quit age, *y*_*quit*_:

$$n\left(y\right)=\{\begin{array}{cc} n_{0}, & y\leq y_{quit}\\ \frac{n_{0}}{1+\gamma \left(y-y_{quit}\right)}, & y>y_{quit} \end{array},$$ (3)

where *γ* is a scaling constant and *n*_0_ is the smoking intensity prior to quitting. *O*_*R*_ quantifies how smoking modulates the baseline risk of dying from lung cancer in those who never smoke, *R*_0_(*y*), modeled by an exponential function with scaling parameters *a* and *b*:

$$R_{0}\left(y\right)=a\text{exp}\left(by\right).$$ (4)

Combining Eqs 2 and 4, the risk of dying from lung cancer, *R*_*death*_, as a function of age (*y*) is

$$R_{death}\left(y\right)=a\text{exp}\left(by\right)\left[1+\beta \frac{n\left(y\right)}{20}\left(y-y_{start}\right)\text{exp}\left(\varphi_{1}\text{ln}\left(n\left(y\right)\right)+\varphi_{2}{\text{ln}}^{2}\left(n\left(y\right)\right)\right)\right],$$ (5)

where *y*_*start*_ is the age at which smoking started. Finally, the free parameters *γ*, *a*, *b*, *β*, *φ*_1_, and *φ*_2_ were determined by fitting the predictions of Eqs 3 and 5 to the mean predictions of cancer death versus age provided by the Cancer Intervention Surveillance and Modeling Network (CISNET) models [15]. The fitting was performed by grid search for 7 different smoking histories simultaneously.

To predict the efficacy of CT screening for lung cancer, what we need to calculate is not the risk of dying Eq (5) but rather the probability, *P*_1_, of getting a first cancerous cell that will eventually grow into a detectable tumor according to Eq 1. Assuming an average tumor burden at death of 500 ml, or 5 × 10^11^ cells [12,16], gives an average time delay, *t*_*delay*_, in years between the appearance of a first cancerous cell and death of

$$t_{delay}=\frac{\text{ln}\left(5\right)+11\;\text{ln}\left(10\right)T_{d}}{365\text{ln}\left(2\right)}.$$ (6)

The probability, *P*_*1*_(*y*), of acquiring a first cancer cell as a function of age and smoking history is thus

$$\begin{array}{ll} P_{1}\left(y\right) & =a\text{exp}\left(b\left(y+t_{delay}\right)\right)\\ & \times \left[1+\beta \frac{n\left(y\right)}{20}\left(y-y_{start}+t_{delay}\right)\text{exp}\left(\varphi_{1}\text{ln}\left(n\left(y\right)\right)+\varphi_{2}{\text{ln}}^{2}\left(n\left(y\right)\right)\right)\right] \end{array}.$$ (7)

Whenever an individual develops an ILC we assume that they immediately gain a fixed probability, *P*_2_, per year of developing a MLC. Once the first cell of this MLC appears it grows at the same rate as the ILC.

### Generation of a screening population

We followed a population of 100,000 individuals with smoking history and tumor growth rate distributions as specified in Table 1 year-by-year beginning at *y* = 1 when they were all cancer free and ending at the age when they were randomized into the screening population. At each age *y* for each individual, we drew a random number, *x*_1_, uniformly distributed on the interval [0, 1]. If *x*_1_ < *P*_1_(*y*) Eq (7), then the individual was designated as having a single tumor cell, and the volume of that tumor was calculated for each subsequent year according to Eq 1. In addition, for each year following the development of ILC another random number, *x*_2_, was drawn. If *x*_2_ < *P*_2_, the individual was designated as having a single MLC tumor cell, which also grew each subsequent year according to Eq 1. Because *P*_1_(*y*) given by Eq 7 is always rather small, even for aged heavy smokers, most of this population was cancer-free. The remainder had either one or two primary lung tumors of various sizes. The age distribution of the population (between the specified limits *y*_1_ and *y*_2_) at the time of the first screening corresponded to the that of the National Lung Screening Trial (NLST) for lung cancer [17]. This study recruited subjects with a frequency, *f*, of ages that decreased approximately linearly with age to about 80 years. The equation for the linear fit to these data, forced to be zero at age 80, is

f(y) = −46y + 3687. (8)

To generate the age distribution we drew a random number, *x*, uniformly distributed on the interval [0, 1]. Because a given value of *x* drawn with probability *P*(*x*) corresponds to an age *y* with probability given by Eq 8, we have the condition

$$\frac{\int_{0}^{x}P\left(x\right)dx}{\int_{0}^{1}P\left(x\right)dx}=\frac{\int_{y_{1}}^{y}f\left(y\right)dy}{\int_{y_{1}}^{y_{2}}f\left(y\right)dy}.$$ (9)

The left-hand side of Eq 9 is simply *x*, and the right-hand side is determined by evaluating the appropriate integrals of Eq 8, which yields the quadratic equation in *y*,

$$y^{2}-160.3y+x\left(\left(y_{1}^{2}-y_{2}^{2}\right)+160.3\left(y_{2}-y_{1}\right)\right)-y_{1}^{2}+160.3y_{1}.$$ (10)

The age for a randomly drawn *x* is the solution to Eq 10 with the negative discriminant root.

### Estimation of tumor detection by low-dose CT

When *V*_*tumor*_ becomes large enough it either kills its host or elicits overt symptoms that lead to medical intervention. In either case, such an individual is not a candidate for screening because the presence of cancer is already evident. There is, however, no precise value of *V*_*tumor*_ at which this occurs; detection of a tumor is a stochastic event. Therefore, we assumed that there is a maximum tumor volume, *V*_max_, estimated to be 1000 ml, above which either death or symptoms are certain to occur; that there is a minimum tumor volume, *V*_min_, set at 100 ml, below which neither death nor symptoms can possibly occur; and that between these two extremes the probability of both changes linearly. Individuals with *V*_*tumor*_ > *V*_max_ were culled from the population while those with *V*_min_ < *V*_*tumor*_ < *V*_max_ were culled if the draw of another random number, *x*_3_, led to the condition. *x*_3_ > (*V*_*tumor*_ − *V*_*min*_)/(*V*_*max*_ − *V*_*min*_). These conditions were imposed for both ILC and MLC.

Individuals remaining in the population at their screening age were subjected to low-dose CT imaging. A tumor with diameter greater than or equal to 4 mm was defined as a positive screen, based on the NLST criteria. If an ILC was detected at the time of first screening before it had reached the limit of resectable volume, *V*_*resect*_, set at 100 ml, it was assumed to have been successfully resected with curative intent. Individuals with *V*_*tumor*_ > *V*_*resect*_ were culled from the population. The lung cancer survivors as well as all other individuals not yet removed from the population were then followed for a specified number of years, *y*_*screen*_, past the time of the initial screening, during which time their tumors were allowed to appear and grow as before. At the first and second screenings, we identified individuals who had died from their tumor burden, individuals whose tumors remained undetected, those whose tumors were detected by CT and were either resectable or not, and those who remained cancer free.

## Results

### Mortality predictions

**Fig 1:**
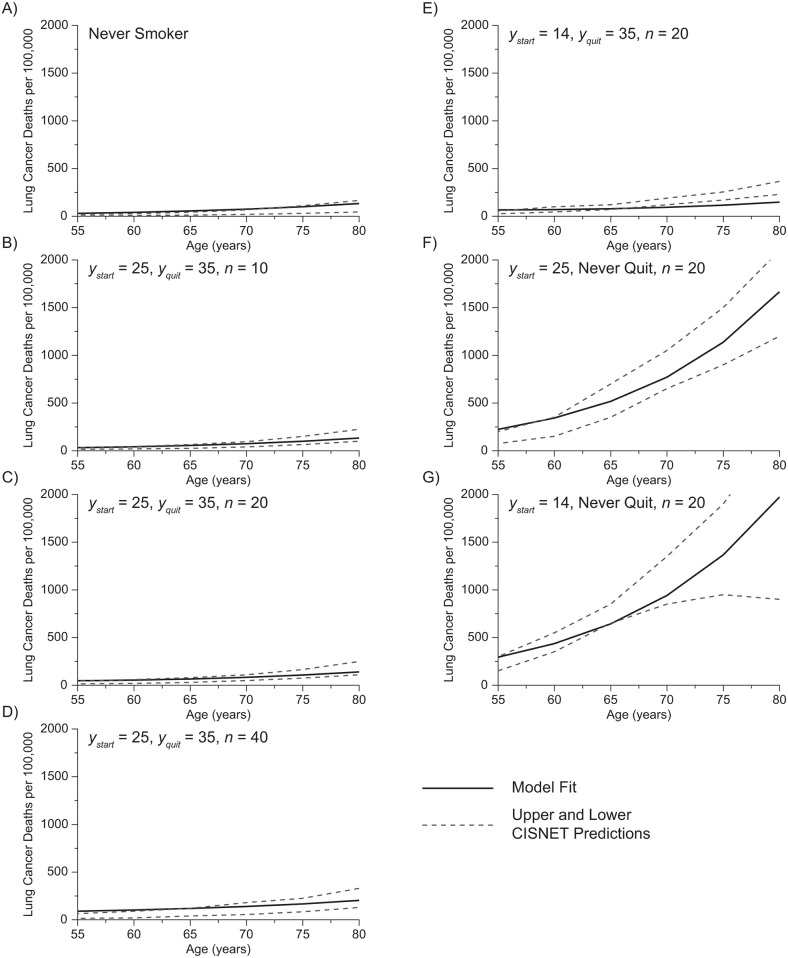
Lung Cancer Mortality Predictions. Each panel shows the number of deaths as a function of age for 7 smoking histories defined by start age, *y*_*start*_, quit age, *y*_*quit*_, and cigarettes per day, *n*. The model fits (solid lines) are enveloped by the upper and lower predictions of the Cancer Intervention Surveillance and Modeling Network (CISNET) models (dashed lines).

Fig 1 shows the predictions of cancer mortality between 55 and 80 years of age provided by Eqs 3 and 5 when the model was fit to the mean predictions provided by the published CISNET models [15]. The 7 different smoking histories in Fig 1 are the same that were used to evaluate the CISNET models. Four of the smoking histories (B–E) include quitting at age 35 years and have predicted mortalities that, while greater than for the never smoker (A), remain quite low. Two of the histories (F and G) involve a lifetime of smoking and accordingly have much higher predicted mortalities. Also shown in Fig 1 are the upper and lower limits of the CISNET model predictions themselves. The fits of Eqs 3 and 5 are either within these limits or close to them and importantly are within the CISNET model limits for the two high-risk scenarios of lifetime smoking. The values of the free parameters in Eqs 3 and 5 providing the fits shown in Fig 1 are *γ* = 0.227, *a* = 1.21 × 10^−5^, *b* = 5.88 × 10^−2^, *β* = 7.5 × 10^−3^, *φ*_1_ = 4.2, and *φ*_2_ = −1.03. The root mean squared residual for these fits is 60 deaths per 100,000 individuals.

### Predictions of lung cancer incidence and CT detection

Table 2 shows incidences in the screened population (excluding those who died prior to the initial screening) of ILC and synchronous lung cancer (SLC, two tumors present) at the initial CT screening and of ILC, SLC, MLC, and death at the time of the second screening (mean (SD) for 16 independent populations of 100,000 individuals with the same demographic and tumor statistics). Individuals whose detected tumors were larger than *V*_*resect*_ populate the “Not Resectable” group and did not receive a second screening, along with those who had both tumors resected. Notably, the rate of CT detection of ILC at the initial screening is comparable to that reported from the NLST (4.5% vs. 3.8%, respectively) [18].

**Table 2:** Cancer Status of Population of Heavy Smokers Aged 55–75 Years at Initial CT Screening and at Follow-up Screenings at Either 1, 3, 5, or 10 Years.

% of Initially Screened Population^a^^,^^b^	Initial CT Screening	Follow-up CT Screening (Years after Initial Screening)			
		1	3	5	10
% Cancer Free	78.37 (0.13)	76.54 (0.13)	72.56 (0.10)	68.29 (0.19)	56.39 (0.13)
ILC only					
% Undetected	13.26 (0.10)	14.00 (0.13)	15.57 (0.11)	17.09 (0.14)	21.03 (0.13)
% Resected	2.03 (0.04)	0.50 (0.02)	1.53 (0.03)	2.49 (0.05)	3.75 (0.06)
% Not Resectable	0.58 (0.03)^d^	0.00 (0.00)	0.01 (0.00)	0.14 (0.01)	1.11 (0.03)
% Dead^c^		0.00 (0.00)	0.01 (0.00)	0.11 (0.01)	3.08 (0.06)
SLC					
% Both Undetected	3.82 (0.05)	4.05 (0.05)	4.53 (0.07)	5.06 (0.09)	6.42 (0.09)
% 1 Resected and 1 Undetected	1.45 (0.04)	0.34 (0.02)	1.04 (0.04)	1.74 (0.04)	2.72 (0.04)
% Both Resected	0.48 (0.02)^d^	0.04 (0.01)	0.21 (0.02)	0.51 (0.02)	0.91 (0.03)
MLC only^e^					
% Undetected		1.40 (0.04)	1.29 (0.04)	1.20 (0.03)	0.96 (0.03)
% Resected		0.14 (0.01)	0.40 (0.02)	0.61 (0.03)	0.64 (0.03)
% Not Resectable		0.00 (0.00)	0.00 (0.00)	0.01 (0.00)	0.14 (0.01)
% Dead^c^		0.00 (0.00)	0.00 (0.00)	0.01 (0.00)	0.42 (0.02)
% No MLC after ILC resection^e^		1.95 (0.04)	1.78 (0.06)	1.64 (0.04)	1.34 (0.03)

ILC: Index Lung Cancer, SLC: Synchronous Lung Cancer, MLC: Metachronous Lung Cancer.

^a^ Mean (SD) of 16 populations with same demographics.

^b^ 5.98% (0.08%) of the initial population died of LC prior to initial screening.

^c^ This represents death from ILC or MLC after the initial screening.

^d^ These groups did not receive follow-up screening.

^e^ This is the population with resected ILC at initial screening.

**Fig 2:**
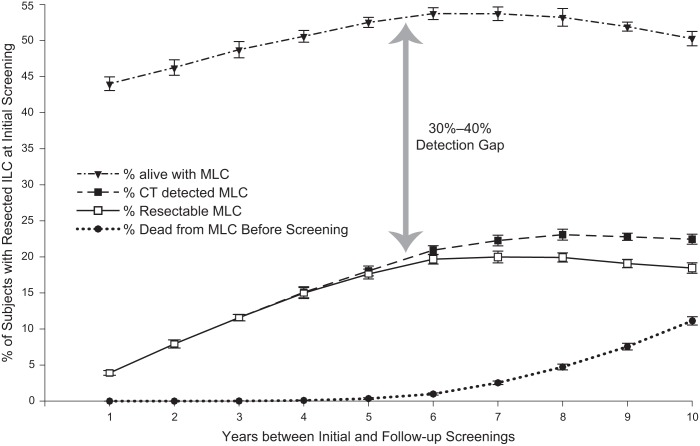
Predictions of Metachronous Lung Cancer (MLC) as a Function Follow-Up Screening Time. The MLC incidence, detection, resection, and mortality is plotted for the subset of subjects who had a resected index lung cancer (ILC) at the initial screening.

Fig 2 demonstrates the model predictions for MLC incidence, detection, resection, and death in the ILC survivor population (3.5% of the initially screened population) as a function of the number of years between the first and second screenings. Several features of these predictions stand out. First, the fraction of individuals with a MLC tumor of any size is already high (> 40%) at 1 year as a result of tumors that develop prior to the initial screening but are too small to be consequential at the time. Second, a large detection gap (30%–40%) separates those with detectable tumors from those with tumors that are too small to detect by CT, highlighting the diagnostic limitations of current high-resolution CT. Finally, our model predicts that virtually all newly detected MLC tumors are small enough to be resectable up to about 5 years after resection of the ILC but that the risk of dying of a potentially preventable MLC (e.g., CT diagnosed and resectable) increases dramatically after that point.

### Model Sensitivity

**Fig 3:**
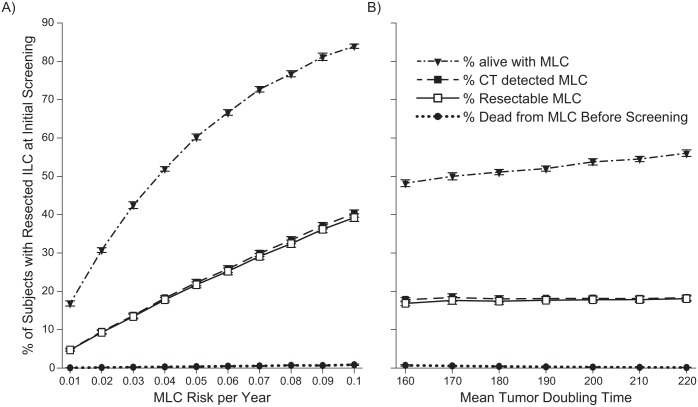
Prediction of Metachronous Lung Cancer (MLC) at 5-Year Follow-Up Screening. The MLC incidence, detection, resection, and mortality is plotted for the subset of subjects who had a resected index lung cancer (ILC) at the initial screening as a function of risk per year of developing MLC (A) and mean tumor doubling time (B).

The predictions shown in Fig 2 were made using a fixed risk per year of developing MLC of *P*_2_ = 4%. This is a nominal value based on somewhat limited data in the literature so we tested the sensitivity of our predictions to this fixed risk. We determined the rates of MLC incidence, detection, resection, and death at 5 years post initial screening in the subset of subjects who had a resected ILC at the initial screening as a function of *P*_2_ between 1% and 10%. These predictions are shown in Fig 3A and demonstrate, not surprisingly, that incidence, detection, and resection of MLC are strongly and increasingly dependent on *P*_2_, while *P*_2_ has little effect on MLC mortality during the post-initial screening period. However, although the difference between the detection and resection rates increases slightly over the range 1% < *P*_2_ < 10%, it remains small compared to the actual rates themselves.

Finally, we tested the sensitivity of our predictions to how rapidly the tumors grow since this obviously has a major direct effect on how rapidly cancers kill their hosts. Fig 3B shows the predicted rates of MLC incidence, detection, resection, and death at 5 years post initial screening in the subset of subjects who had a resected ILC at the initial screening as a function of mean tumor doubling time between 160 and 220 days. Interestingly, the predicted quantities are rather static over this range, no doubt because of the existence of a range of mean tumor doubling times already in the population. That is, the initial screening automatically selects into the ILC survivor group those members of the population who have the fixed subset of tumor doubling times that give rise to resectable MLC tumors at the time of screening. We also evaluated maximum tumor volumes that would result in death or symptoms, varying this value from 700 mL to 1300 mL. This did not result in any difference in rates of death or unresectable tumors at 5 years.

## Discussion

ILC survivors are at a higher risk of developing new lung cancer than the smoking population as a whole, likely reflecting the fact that the occurrence of MLC selects out those individuals with a history of other carcinogenic exposures and/or a genetic propensity to develop lung cancer in the first place. Identifying MLC is important because, when identified early, it is associated with good outcomes.[5,6] Nevertheless, the strategy for following patients at risk for MLC remains unclear. Accordingly, our goal in the present study was to establish a predictive computational model to aid the rational design of screening strategies for patients whose ILC has been resected with curative intent. Confirmation of the usefulness of any such strategy can only be gained through clinical trials, but the cost of such investigations can be prohibitive.

A substantial amount of epidemiological data exists concerning ILC, which have been encapsulated computationally in the CISNET models that predict risk values for death as a function of age and smoking history [15]. These models serve as the evidence base for the current US Preventive Services Task Force recommendations for lung cancer screening [8]. Nevertheless, the CISNET models exhibit a significant degree of spread in their individual predictions (Fig 1). We therefore reasoned that our model would provide a well-validated basis upon which to predict the risk of ILC occurrence if its predictions of death were fit to the means of the CISNET predictions and fell within the CISNET predictions bounds. The estimates produced by our model were found to lie within the CISNET prediction bounds except for some instances of modest smoking history (Fig 1) that do not have a substantial effect on the accuracy of overall predictions for a population in which cancer tends to occur in individuals with the most extensive smoking histories. Although serving as the basis for current screening guideline, the epidemiologic data upon which the CISNET models and our computational model are based are not derived from studies of CT screening. However, our predictions of CT screening efficacy (Table 2) are also comparable to those reported from clinical trials, most notably the NLST [7].

Building on a well-grounded prediction of the incidence of ILC we then incorporated a predictive model for MLC. We felt it was epidemiologically important to include an initial screening population, rather than merely starting from a population with treated lung cancers for several reasons. There are few data regarding the risk of MLC in a population of smokers that underwent CT screening for an ILC. Tumor biology presumably partially selects the risk set for second lung cancer by culling those individuals with tumors that grow very slowly and are too small to be detected and those with tumors so aggressive that the patient would die from the tumor prior to screening. MLC is defined clinically based on temporal occurrence, histology, and location [4]. Because we calculate the rate of growth of tumors that are too small to be detected by CT, the model allows us to accurately distinguish MLC from ILC, thus eliminating the need for the more complicated clinical definition. Our model does not include the risk of recurrent lung cancer, allowing us to estimate the pure effect of MLC. However, because the model is designed to estimate the isolated effect of MLC and does not include a risk of recurrent ILC, making comparisons with clinical data is somewhat uncertain. We partially addressed this by assessing the sensitivity of the model to changes in the probability of MLC. A risk of 4% per year represents a median estimate based on reported numbers in the literature [3,4,11,19,20]. Varying the probability from 1% to 10% did not result in significant differences in the performance of screening (percent of resectable MLC detected by CT versus the percent dead before screening for MLC).

The results of our modeling study suggest that, with current CT screening resolution, screening for MLC should continue well after 5 years following treatment of the ILC since we continue to detect potentially resectable cancers that would increase mortality if not treated. This risk approaches 10% by 10 years (Fig 2) and does not appear to diminish over time. This 5-year time period varies little with variations in *P*_2_ between 1% and 10% (Fig 3A) and with variations in mean tumor doubling time between 160 and 220 days (Fig 3B). Lung cancer survivors in general are scanned at least yearly [9,10]; however, the number of years over which this practice should be continued remains unknown. Our results highlight that the risk of potentially preventable death (e.g., CT detection followed by resection) from MLC increases dramatically after the first five years following surgical resection of an ILC, implying that there is no safe time to stop CT screening. Parenthetically, the five-year mark is classically recognized as the point where a lung cancer is more likely to be a second primary and not a recurrence.

An unexpected finding of our predictive model is the “detection gap” of 30%–40% in the 10 years following ILC resection due to those subjects who have undetectable MLC. Although this estimate certainly includes cancers that a patient would have died with, not necessarily of (e.g., overdiagnosis), the entirety of the diagnostic gap seems unlikely to be accounted for by this explanation. Estimates of overdiagnosis in lung cancer screening generally range from 10% to 12% with an absolute upper bound of 35% [21]. Thus, despite recent advances in CT imaging technology there remains substantial potential to save lives with improvements in tumor detection such as could be provided, for example, by a cancer biomarker sensitive to the presence of fewer malignant cells than are detectable by CT scanning.

These modeling results, however, were obtained in an ideal setting that is not complicated by the various factors that bedevil cancer screening. Probably most important of these is the very high false positive rate for lung tumor detection. Roughly 19 in 20 suspicious findings on CT turn out not to be cancer [18], and we have not considered the substantial morbidity associated with the investigation of every finding. We also did not consider the occurrence of false negatives, which would reduce the incidence of lung cancer resection with curative intent and allow the undetected cancers to proceed to a more lethal stage. Risks of death from causes other than lung cancer were not included in the model because we focused on the key issues necessary to estimate a maximal effect of MLC. However, there is a potential that the model has underestimated the potential benefit of CT screening since cancer risk is only based on age and smoking history. If additional risk factors such as concomitant lung disease, family history of lung cancer, radon or occupational exposures, for instance, substantially increase the risk of MLC then CT screening may have further benefit. All of these neglected factors certainly have the potential to affect detection rates and survival to a substantial degree. What we have simulated in the present study thus amounts to a maximal impact of second lung cancers on mortality, based on known risk factors, and the largest potential effect of CT screening for these cancers at a given detection size (here 4 mm). Our model does not address the possibility that the natural history of screen detected lung cancer may be significantly different from the data used to inform the CISNET models which was acquired from symptomatic or incidentally detected lung cancers. However, long term-follow up data in large populations who have undergone CT screening is not currently available. As this data becomes available, our model may need to be adapted for a potentially different lung cancer natural history. Similarly, the predictions of our model are contingent upon the numerous assumptions that were made regarding cancer biology that include an exponential tumor growth unlimited by changing nutrient availability or genetic degradation, identical growth rates for both ILC and MLC, and a probability of survival that is purely a function of tumor size. Certainly there is wide variation in lung tumor growth rates and our model does not capture of all this heterogeneity. It is also particularly worth noting that this model may be less representative of pure ground glass lesions, which are known to have a tumor doubling time greater than 400 days. The assumptions we have made largely represent first approximations to what are in many cases much more complicated situations, and our predictions must be viewed accordingly. Confirmation of the usefulness of CT screening for MLC may only be gained through prospective clinical trials. However, the cost of these investigations can be prohibitive, implying a role for iterative improvement in computational estimations of the benefits of such a screening intervention prior to undertaking a trial.

In summary, we have constructed a predictive model of MLC occurrence and detection by CT in patients who have had a successfully resected ILC. The model is anchored to extensive epidemiological data on lung cancer incidence and detection that allow predictions of MLC incidence, detection, and death. Applying the model to a hypothetical high-risk population suggests that screening for MLC within 5 years after resection of an ILC may identify only a very small number of treatable cancers but that continuing to screen after this point is imperative given the dramatic increase in risk of death from potentially detectable MLC. The model also predicts a substantial detection gap for MLC that demonstrates the potential benefit to be gained as more sensitive screening methods are developed.

## Abbreviations

CISNET: Cancer Intervention Surveillance and Modeling Network
ILC: index lung cancer
MLC: metachronous lung cancer
NLST: National Lung Screening Trial
SLC: synchronous lung cancer
